# Longitudinal associations between physical activity and preferred walking speed in community-dwelling older adults: a 4-year follow-up study

**DOI:** 10.1186/s11556-026-00419-9

**Published:** 2026-06-04

**Authors:** Joona Neuvonen, Matti Hyvärinen, Antti Löppönen, Timo Aittokoski, Taina Rantanen, Laura Karavirta

**Affiliations:** Gerontology Research Center and Faculty of Sport and Health Sciences, University of Jyväskylä, Jyväskylä, Finland

**Keywords:** Wearable devices, Physical activity energy expenditure, Mobility, Aging

## Abstract

**Background:**

The ability to engage in physical activity reflects preserved physical function, autonomy, and well-being in older adults, making physical activity a key outcome of healthy aging. Walking is a common form of physical activity in later life, so understanding how walking speed is associated with overall activity is particularly important. This study examined 4‑year changes in physical activity energy expenditure (PAEE) in community‑dwelling older adults and assessed preferred walking speed in relation to PAEE.

**Methods:**

This study used data from AGNES, a 4-year follow-up study of initially 75-, 80-, and 85-year-old adults. PA was measured using thigh-worn accelerometers and ECG monitors, with PAEE estimated using a branched equation model integrating heart rate and acceleration data. Preferred walking speed was measured using a self-paced six-minute walk test. Associations between PAEE, walking speed, and their 4-year changes were examined using linear mixed models.

**Results:**

Among 299 participants, PAEE declined by 16.9 kJ·kg^− 1^·day^− 1^ over 4 years. Preferred walking speed showed a positive association with PAEE, with each 1 km·h^− 1^ higher speed corresponding to 4.7 kJ·kg^− 1^·day^− 1^ higher PAEE. There was limited evidence for an association between changes in preferred walking speed and changes in PAEE over the 4‑year follow‑up period.

**Conclusions:**

In community-dwelling older adults, PAEE declined substantially with age, more steeply than in total physical activity previously reported in similar cohorts. Walking speed was strongly associated with PAEE, but its change was not consistently associated with changes in PAEE.

**Supplementary Information:**

The online version contains supplementary material available at 10.1186/s11556-026-00419-9.

## Introduction

Physical activity (PA), defined as any bodily movement produced by skeletal muscles that results in energy expenditure [1], generally declines with age [2], with the steepest reductions typically observed in adults over 70 years [3]. Globally, a substantial proportion of older adults fail to meet the World Health Organization’s recommendations for PA, which include at least 150 min of moderate-to-vigorous-intensity activity per week [4, 5]. This shortfall has significant implications. Insufficient PA is associated with increased risk of chronic diseases, functional decline, loss of independence, and premature mortality [6–8].

Despite the wide range of activities older adults may engage in, walking remains the most common and accessible form of PA in this age group [9, 10]. For many older adults, walking represents a major component of their physical activity-related energy expenditure (PAEE) [11]. Therefore, longitudinal changes in the absolute intensity of walking bouts may influence the total PAEE accumulated under free-living conditions in older adults through potential effects of slower walking speed on wearable-derived estimates of activity intensity. As such, understanding how preferred walking speed/intensity changes with age, and how it relates to overall PAEE, is critical for identifying early signs of functional decline and for designing effective interventions to promote healthy and active aging.

Age-related changes in preferred walking speed, a key indicator of physical function, may reflect broader age‑related processes associated with lower PAEE. Longitudinal studies have shown that reductions in preferred walking speed are associated with decreases in PA over time, both on self-reported measures [12] and on accelerometer-based estimates [13]. However, despite clear evidence that the decline in preferred walking speed accelerates in advanced age, no longitudinal studies have directly examined adults aged 75 years and older. Specifically, it remains unclear how changes in walking speed relate to changes in total activity measured using wearable sensors in older adults at ages where walking speed decline is most pronounced [14].

Because PAEE derived from wearable sensors may be influenced by age-related changes in walking speed, interpretation of walking speed–PAEE relationships requires careful consideration. Wearable sensors are typically calibrated for assessing PAEE using laboratory-based protocols, where participants perform standardized activities e.g., walking at various speeds [15]. Accelerometer algorithms infer activity intensity from the vector magnitude of acceleration; therefore, age-related reductions in walking speed in older adults may be interpreted as reduced activity intensity and volume, even when similar activities are performed for the same duration [16]. This may lead to lower estimates of PAEE over time that partially reflect changes in walking rather than true declines in physical behaviour. Although incorporating heart rate (HR), which provides a physiological signal of energy expenditure complementary to acceleration, may help mitigate some of these limitations in estimation accuracy [17, 18], the extent to which wearable-derived PAEE reflects age-related changes in walking speed remains unclear. Understanding how wearable-derived metrics respond to age-related changes in walking speed is therefore essential for accurately interpreting longitudinal activity patterns in later life.

In this study, we examine longitudinal changes in PAEE and evaluate preferred walking speed as a potential predictor of change. In addition to longitudinal associations, we also assess how much of the cross-sectional variation in PAEE is explained by preferred walking speed. Specifically, we ask the following research questions: (1) How much does PAEE change over a 4-year period in community-dwelling older adults? (2) Is preferred walking speed associated with PAEE during the follow-up period? (3) Do individual changes in preferred walking speed correspond to longitudinal changes in PAEE over the 4-year period?

## Methods

This longitudinal study utilized data from the population-based study "Active Ageing – Resilience and External Support as Modifiers of the Disablement Outcome" (AGNES), which involved three age cohorts of initially 75-, 80-, and 85-year-old men and women. The participants were drawn from a population register and included individuals living independently in Jyväskylä, Finland. Follow-up data were collected during 2021–2022, following the protocol established for the baseline assessments in 2017–2018 [19].

This study utilized various data collection methods, including a postal questionnaire, a home interview, PA monitoring, and a laboratory visit. Of the 495 participants who consented to PA monitoring at baseline [20], 45 participants lost interest during the follow-up period, 34 were deceased, 8 were excluded due to health reasons, and 11 were unreachable. This left 397 participants eligible for follow-up. Of these, 308 consented to participate in the follow-up assessments. Among those, 299 participants provided valid PA monitoring data at both baseline and follow-up.

### Preferred walking speed

Preferred walking speed was assessed using a laboratory-based, modified six-minute walk test (6MWT). During the test, participants were instructed to walk at their usual, comfortable pace along a 20-meter corridor, turning at each end, for a total duration of six minutes. The walking was followed by two minutes of seated recovery [19]. Rate of perceived exertion (RPE) was assessed using the Borg 6–20 scale [21].

Preferred walking speed was calculated by dividing the total distance walked during the test by duration (6 min). Preferred walking speeds are presented as kilometers per hour (km·h^− 1^). Only complete tests, where participants walked for the full six minutes without interruption, were included in the analyses involving preferred walking speed.

### PA monitoring

#### Accelerometry

Participants were instructed to wear a tri-axial accelerometer (100 Hz, 13-bit, ± 16 g, UKK RM42, UKK Terveyspalvelut Oy, Tampere, Finland) continuously (24 h) for seven days at baseline and five days at the follow-up, secured with waterproof self-adhesive film on the thigh of the dominant leg. The raw acceleration data were calibrated to local gravity using an optimization method that adjusted gain and offset [22, 23]. Then, the mean amplitude deviation (MAD) was calculated from the vector magnitude of the acceleration in non-overlapping 5-second intervals [24]. MADs were finally resampled into 10-second intervals to align with HR time series. At least three full valid days were required at both time points for participants to be included in final analyses.

#### Heart rate

Simultaneously with the thigh accelerometer, participants wore a single-channel chest electrocardiogram (ECG) monitor (250 Hz, eMotion Faros 180, Bittium Corporation, Oulu, Finland). The monitor was positioned either on the sternum or diagonally on the left side below the breast. At baseline, the ECG monitor was renewed once during the monitoring period, resulting in approximately 7 days of ECG recordings. At follow-up, the monitor was worn continuously until the battery was depleted, typically after 4 days.

R-peak detection was performed using a custom signal processing pipeline in MATLAB R2024b (The MathWorks, Inc., Natick, MA, USA). The primary detection method included several preprocessing steps: the ECG signal was filtered using a low-pass filter (62.5 Hz) to remove high-frequency noise and a high-pass filter (24.4 Hz) to eliminate baseline drift. The filtered signal was then differentiated to emphasize QRS slopes, squared to enhance large signal changes, and normalized to its maximum absolute amplitude to standardize the signal. A moving average filter (0.125 s) was applied twice to smooth the signal and highlight QRS complexes. To improve sensitivity and recover missed peaks, a secondary detection pass was performed using the algorithm by Rodrigues et al. [25]. Any peak identified by this algorithm that was not detected in the primary pass within a ± 0.2-second tolerance window (i.e., 200 ms around the primary peak location) was added to the final set of R-peaks. Prior to HR calculation, ECG data were quality-filtered using a deep learning model trained to annotate signal quality. Training data was generated via a semi-automated annotation pipeline that compared the presence of R-peaks detected by the Rodrigues et al. [25] algorithm against expert-labeled R-peaks in ECG recordings. R-peaks were manually annotated to a laboratory dataset of 59 ECG recordings (19 min each: 5 min supine, 6 min standing, 6 min walking, 2 min sitting) by an exercise physiologist using Kubios HRV Premium 3.2.0 (Kubios Oy, Kuopio, Finland). R-peaks matching expert annotations were assigned the quality value of 1 and unmatched peaks were assigned 0.

A one-dimensional convolutional neural network (1D CNN) was built using MATLAB’s Deep Learning Toolbox to assign a quality score (0–1) to each detected R-peak based on the training dataset. The network architecture included a sequence input layer, two convolutional layers with causal padding (filter size = 64), ReLU activations, layer normalization, a global average pooling layer, a fully connected layer, softmax activation, and a classification output layer. The model was trained using the Adam optimizer (learning rate = 0.001) for 160 epochs, with data split into training (50%), validation (20%), and test (30%) sets. The best-performing model achieved 94.7% classification accuracy and was used to annotate all free-living R-peaks. ECG segments with quality scores below 0.5 were excluded from HR calculation. Similar to ACC, HR was calculated in 10-second epochs from the RR time series.

### Physical activity energy expenditure

Physical activity energy expenditure (PAEE) in free-living conditions was estimated using a combination of accelerometry (ACC) and HR data. Initially, PAEE was calculated independently from ACC and HR and then these two estimates were then integrated using a branched equation model. ACC-based PAEE was calculated from MAD values using a linear equation derived from indirect calorimetry during treadmill walking in a sample of older adults of similar age, as described by Karavirta et al. [20]:

$$\:{\mathrm{P}\mathrm{A}\mathrm{E}\mathrm{E}}_{ACC}\:\left(\mathrm{J}\cdot\:{\mathrm{k}\mathrm{g}}^{-1}\cdot\:{\mathrm{m}\mathrm{i}\mathrm{n}}^{-1}\:\right)=\:295.3\:\mathrm{*}\:\mathrm{M}\mathrm{A}\mathrm{D}\:+\:90.8$$

This method was applied throughout the entire monitoring period, independent of HR data availability. HR-based PAEE was calculated using an individual walk test calibration protocol as described by Westgate et al. [26]. Calibration was performed at the individual level and included: sex (coded 0 = woman, 1 = man), sleeping heart rate (HR_sleep_, defined as the average of the 10 lowest nightly HR values from free-living data), walking HR (HRaS_walk_, average HR above sleeping HR during the final minute of the 6MWT), recovery heart rate (HRaS_rec_, HR above sleeping HR at 45 s post-walk), walking speed (m·s^− 1^) from the 6MWT, beta-blocker status (coded 0 = no, 1 = yes) from the postal questionnaire, and body height (in meters) measured during the laboratory visit. Flex heart rate (Flex HR) was defined as the average of HR at a PAEE of 108 J·kg^−^¹·min^−^¹ and sleeping HR + 10 bpm, with PAEE set to 0 for HR values below this point [20]. PAEE during free-living conditions was then estimated from heart rate above HR_sleep_ (HRaS) using this equation with added weightings to parameters similar to Karavirta et al. [20].

For participants who had free-living HR monitoring but either did not complete the 6MWT or had undetermined HRs from the 6MWT (*n* = 46), a group-calibration equation was used. This was done by fitting calibration factors, including age (in years) to the intercepts and slopes derived from participants who had individual calibration data available. Both individual- and group-level calibrations were performed separately for each time point, using data collected at that specific time point. Separate calibration equations were used at baseline and follow‑up to ensure that PAEE estimates reflected relationships that vary with age and medication status at each time point. Resulting group calibration equations were, for baseline:

$$\begin{aligned}\:{\mathrm{P}\mathrm{A}\mathrm{E}\mathrm{E}}_{HR}\:(\mathrm{J}\cdot\:{\mathrm{k}\mathrm{g}}^{-1}\cdot\:{\mathrm{m}\mathrm{i}\mathrm{n}}^{-1}\:)\;&=\;(7.1640\:-\:0.0080\:*\:age\:+\:0.3724\:*\:sex\:-\:0.0001\:*\:{HR}_{sleep}\;\\&+\;0.0826\:*\:betablocker)\:*\:\mathrm{H}\mathrm{R}\mathrm{a}\mathrm{S}\:+\:(-1.9358\:*\:age\:+\:21.8186\:*\:sex\;\\&-\;0.1059\:*\:{HR}_{sleep}\:\:+\:63.9324\:*\:betablocker\:-\:16.9634)\end{aligned}$$

, and for follow-up:

$$\begin{aligned}\:{\mathrm{P}\mathrm{A}\mathrm{E}\mathrm{E}}_{HR}\:(\mathrm{J}\cdot\:{\mathrm{k}\mathrm{g}}^{-1}\cdot\:{\mathrm{m}\mathrm{i}\mathrm{n}}^{-1}\:)\;&=\;(7.7814\:-\:0.0145\:*\:age\:+\:0.3426\:*\:sex\:-\:0.0021\:*\:{HR}_{sleep}\\&+\:0.0607\:*\:betablocker)\:*\:\mathrm{H}\mathrm{R}\mathrm{a}\mathrm{S}\:+\:(-3.7174\:*\:age\:+\:11.4328\:*\:sex\;\\&-\:0.7292\:*\:{HR}_{sleep}\:\:+\:59.3666\:*\:betablocker\:+\:160.0595)\end{aligned}$$

Finally, PAEE was estimated using a branched equation model that integrates the ACC- and HR- based PAEE developed by Brage et al. [27]. This model dynamically adjusts the weighting of HR and ACC depending on activity intensity. Under typical free-living conditions with little or no movement, ACC is weighted more heavily, while HR receives greater weight during higher-intensity activities. If HR data were unavailable either completely or in part, PAEE was estimated solely from ACC. PAEE is expressed in kilojoules per kilogram per day (kJ·kg^−^¹·day^−^¹).

### Participant characteristics

During the home interview, participants reported the duration of their education in years, which was used as a proxy for socioeconomic status [28]. Self-rated health was evaluated using the question: *“Do you consider your health at the moment to be (1) very good*,* (2) good*,* (3) average*,* (4) poor*,* or (5) very poor?”* [29]. Cognitive function was assessed using the Mini-Mental State Examination (MMSE), and physical function was evaluated with the Short Physical Performance Battery (SPPB) during the home interviews [19].

### Statistical analyses

Changes in participant characteristics, PAEE, and HR-derived calibration parameters used for PAEE estimation from baseline to the 4-year follow-up were assessed using paired, two-tailed Student’s t-tests for continuous variables. Normality of the differences between paired observations were assessed using the Shapiro-Wilk test. Categorical variables were analyzed using McNemar’s test for beta-blocker status and the McNemar-Bowker test for self-rated health. Participant characteristics are presented as means ± standard deviations (SD) for continuous variables and as percentages for categorical variables.

To investigate the predictors of PAEE during the follow-up period, linear mixed models (LMMs) were employed. The primary aim was to assess the associations between walking speed and PAEE during the follow-up period. *Baseline age* was included to capture cross sectional differences associated with chronological age, whereas *time point* distinguished baseline from follow up measurement, and served as a dichotomous variable capturing within person change over the follow up period. Alongside age and time, *preferred walking speed* was added as a potential predictor of PAEE, along with its interaction with time point to assess changes over time. Participant’s study identifier was included as a random effect to account for repeated measures. Additionally, models were adjusted for potential confounding factors. Specifically, LMMs were constructed as follows:

- *Model 1* included baseline age, and time point as fixed effects to estimate PAEE.
- *Model 2* extended Model 1 by adjusting for additional potential confounders in measurement of PA: *sex*, *beta-blocker status*, *accelerometer wear time*, *HR availability* (defined as the percentage of quality-filtered HR data per day), and the interaction between HR availability and time point.
- *Model 3* added preferred walking speed and its interaction with time point to Model 2.
- *Model 4* was constructed as a separate model with a distinct purpose: it replaced preferred walking speed in Model 3 with the *change in preferred walking speed* from baseline to follow-up, added baseline preferred walking speed to isolate the effect of the change, and included an interaction term between the change in walking speed and time point to assess whether changes in walking speed were associated with corresponding changes in PAEE over time (i.e., whether they modified the time-related change in PAEE).

In addition to the primary PAEE estimates based on combined HR and ACC, LMM analyses were repeated using accelerometer-derived PAEE alone as sensitivity analyses to facilitate comparison with previous studies that relied on ACC-only measures of PA.

All variables in the LMMs were mean-centered. Model assumptions were evaluated using residual plots, Q-Q plots and correlation analyses. LMM results are reported as unstandardized regression coefficients (B) with 95% confidence intervals or p-values.

All statistical analyses were conducted using R Statistical Software (v4.5.1; R Core Team, 2021). Statistical significance was set at *p* < 0.05.

## Results

### Participant characteristics

A total of 299 participants (mean age at baseline: 78.0 ± 3.2 years, 57% women) were included in the analyses. Over the 4-year follow-up, height, body mass, and body mass index declined in both men and women. SPPB scores declined by -0.6 points (*p* < 0.001), indicating a reduction in physical function. Self-rated health shifted toward lower categories over time (*p* < 0.001), with the proportion of participants rating their health as “very good” or “good” decreasing from 59% to 42% The proportion reporting “average” health increased from 38% to 54%. Additionally, the proportion reporting “poor” or “very poor” health increased from 2% to 4%. MMSE scores showed no significant change overall. Participant characteristics, stratified by sex and time point, are presented in Table 1.

**Table 1:** Participant characteristics at baseline and 4-year follow-up (*n* = 292–299)

	Total							Men							Women						
	Baseline			4-Year Follow-up				Baseline			4-Year Follow-up				Baseline			4-Year Follow-up			
	*n*	Mean / Count	SD / %	n	Mean / Count	SD / %	*p*	*n*	Mean / Count	SD / %	*n*	Mean / Count	SD / %	*p*	*n*	Mean / Count	SD / %	*n*	Mean / Count	SD / %	*p*
Age (years)	299	78.0	3.2	299	81.9	3.2	< 0.001	129	78.1	3.3	129	82.0	3.3	< 0.001	170	77.9	3.2	170	81.7	3.1	< 0.001
Height (cm)	299	165.0	8.9	287	164.4	9.1	< 0.001	129	172.8	5.9	126	172.2	6.1	< 0.001	170	159.0	5.4	161	158.3	5.7	< 0.001
Body mass (kg)	299	75.3	13.2	287	73.3	12.9	< 0.001	129	81.1	11.9	126	78.6	11.5	< 0.001	170	70.9	12.4	161	69.2	12.4	< 0.001
BMI (kg·m^− 2^)	299	27.7	4.4	287	27.1	4.2	< 0.001	129	27.2	3.8	126	26.5	3.7	< 0.001	170	28.0	4.8	161	27.6	4.6	< 0.001
Education (years)	297	12.1	4.4					127	12.4	4.7					170	11.8	4.1				
SPPB (points)	299	10.6	1.7	298	10.0	2.2	< 0.001	129	10.8	1.6	128	10.5	1.9	0.003	170	10.4	1.8	170	9.6	2.3	< 0.001
MMSE (points)	298	27.7	2.2	299	27.5	2.4	0.113	129	27.4	2.5	129	27.3	2.8	0.619	169	28.0	1.9	170	27.7	2.1	0.074
Self-rated health	299			297			< 0.001	129			128			< 0.001	170			169			0.005
Very good /good		177	59		126	42			85	66		58	45			92	54		68	40	
Average		115	38		159	54			43	33		67	52			72	42		92	54	
Poor / Very poor		7	2		12	4			1	1		3	2			6	4		9	5	

*BMI *body mass index. *P*-values from paired Student’s t-test for baseline vs. follow-up, except for self-rated health (McNemar-Bowker test)

### Physical activity energy expenditure and preferred walking speed

**Fig. 1:**
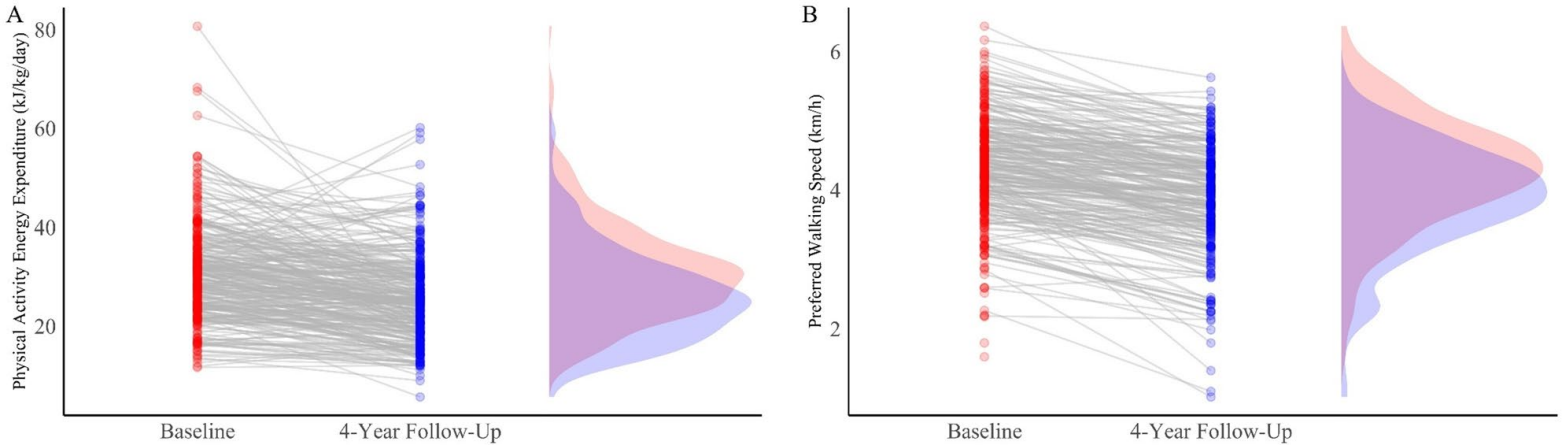
Individual changes and the distribution of PAEE (A, *n* = 299) and preferred walking speed (B, *n* = 264)

Changes in PAEE and HR-based calibration parameters from baseline to the 4-year follow-up are presented in Table 2. On average, PAEE decreased significantly over time (from 31.0 ± 10.0 to 25.7 ± 9.0 kJ·kg^−^¹·day^−^¹, *p* < 0.001). Mean accelerometer wear time was shorter at follow-up compared to baseline, while HR availability per day remained similar. Mean preferred walking speed declined significantly from 4.39 km·h^−^¹·to 3.88 km·h^−^¹ (*p* < 0.001). Individual changes in PAEE and preferred walking speed are presented in Fig. 1.

**Table 2:** PAEE, walking performance and HR-based calibration variables at baseline and 4-year follow-up (*n* = 185–299)

			Baseline		4-year follow-up		
		*n*	Mean / Count	SD / %	Mean / Count	SD / %	*p*
PAEE (kJ·kg^− 1^·day^− 1^)		299	31.0	10.0	25.7	9.0	< 0.001
Wear time (days)		299	7.07	1.10	3.95	0.21	< 0.001
HR availability per day (%)		299	58.9	33.2	59.6	36.3	0.753
Preferred walking speed (km·h^− 1^)		264	4.39	0.71	3.88	0.77	< 0.001
Walking RPE (Borg scale)		264	11.9	2.4	12.4	2.3	< 0.001
Walking HR (bpm above sleep)		186	51.2	14.1	49.5	14.9	0.044
Recovery HR (bpm above sleep)		186	35.8	12.8	34.5	13.5	0.131
Flex HR (bpm above sleep)		185	23.8	5.1	24.9	5.6	< 0.001
Sleeping HR (bpm)		261	52.3	6.7	52.2	7.1	0.841
Slope of HR-PAEE relationship (J·kg^− 1^·beat^− 1^)		185	6.72	0.25	6.66	0.25	< 0.001
Intercept of HR-PAEE relationship (J·min^− 1^·kg^− 1^)		185	-143	61	-154	66	0.002
Beta blocker usage		299					0.584
No			186	62	180	60	
Yes			113	38	119	40	

*PAEE* physical activity energy expenditure, *HR *heart rate, *RPE *rate of perceived exertion. *P*-values derived from paired Student’s t-test, except for beta blocker usage (McNemar’s test)

### Physical activity energy expenditure calibration parameters

Some changes in walking speed and HR responses were observed over time (Table 2). Walking HR decreased, while recovery HR showed no significant change. Flex HR increased over time, and sleeping HR remained unchanged. The proportion of participants using beta blockers remained similar. As a consequence of changes in walking speed and HR, the calibration equation used to estimate PAEE also changed: the slope and the intercept of the HR–PAEE relationship decreased.

### Longitudinal associations of walking speed with physical activity energy expenditure

Associations of age at baseline, time between measurements, preferred walking speed, and potential confounders with PAEE are presented in Table 3.

**Table 3:** LMM estimates of age, time, sex, and preferred walking speed on PAEE (*n* = 282–299)

Independent variables		Estimate	95% confidence interval
Model 1 (Number of observations = 598, *n* = 299)			
(Intercept)		30.9***	[29.9, 32.0]
Baseline age (year)		-0.44**	[-0.73, -0.14]
Time point (reference = baseline)			
Follow-up		-5.21***	[-6.25, -4.17]
Model 2 (Number of observations = 556, *n* = 287)			
(Intercept)		29.1***	[27.7, 30.6]
Baseline age (year)		-0.424**	[-0.701, -0.147]
Time point (reference = baseline)			
Follow-up		-5.25***	[-6.29, -4.21]
Sex (reference = women)			
Men		4.92***	[3.11, 6.73]
Beta blocker (reference = no)			
Yes		-1.31	[-2.94, 0.324]
Wear time (day)		-0.836*	[-1.65, -0.0214]
Heart rate availability (%)		0.0387**	[0.0105, 0.067]
Heart rate availability × Time point		-0.0237	[-0.0577, 0.0101]
Model 3 (Number of observations = 516, *n* = 282)			
(Intercept)		29.5***	[28.2, 30.9]
Baseline age (year)		-0.0295	[-0.301, 0.241]
Time point (reference = baseline)			
Follow-up		-4.63***	[-5.74, -3.51]
Sex (reference = women)			
Men		3.21***	[1.49, 4.93]
Beta blocker (reference = no)			
Yes		0.104	[-1.48, 1.69]
Wear time (day)		-0.696	[-1.51, 0.118]
Heart rate availability (%)		0.0314*	[0.00294, 0.0599]
Preferred walking speed (km/h)		4.73***	[3.44, 6.01]
Heart rate availability × Time point		-0.0229	[-0.0588, 0.0128]
Preferred walking speed × Time point		-0.568	[-2.06, 0.93]

*LMM  *linear mixed model, *PAEE *physical activity energy expenditure. *p ≤ 0.05, **p ≤ 0.01, ***p < 0.001

*PAEE *physical activity energy expenditure

In Model 1, both baseline age and time point were significantly associated with PAEE. Older age at baseline was associated with lower PAEE during the follow-up period, with each additional year of age corresponding to -0.44 kJ·kg^− 1^·day^− 1^ (*p* ≤ 0.01) lower PAEE. PAEE decreased significantly during the follow-up (B = -5.21 kJ·kg^− 1^·day^− 1^, *p* < 0.001), which corresponds to a 17.0% decline in PAEE. Model 2 showed that these associations remained after adjusting for confounders.

In Model 3, preferred walking speed was associated with PAEE across timepoints (B = 4.73 kJ·kg^− 1^·day^− 1^, *p* < 0.001), but its interaction with time point was not. Baseline age was no longer associated with PAEE, whereas time point remained significant (B = -4.63 kJ·kg^− 1^·day^− 1^, *p* < 0.001). In Model 4, the interaction between changes in walking speed and time point was not statistically significant (B = 2.30 *p* = 0.09), indicating no association between changes in walking speed and change in PAEE over time (Table 4).

**Table 4:** LMM estimates of change in preferred walking speed on PAEE (*n* = 252)

Independent variables			Estimate	95% confidence interval
Model 4 (Number of observations = 486, *n* = 252)				
(Intercept)			29.9***	[28.5, 31.3]
Baseline age (year)			-0.0244	[-0,324, 0,275]
Time point (reference = baseline)				
Follow-up			-5.16***	[-6.29, -4.03]
Sex (reference = women)				
Men			2.86**	[1.04, 4.68]
Beta blocker (reference = no)				
Yes			-0.0223	[-1.68, 1.64]
Wear time (day)			-0.654	[-1.48, 0.181]
Heart rate availability (%)			0.0298	[-0.000141, 0.0598]
Baseline preferred walking speed (km·h^− 1^)			4.59***	[3.23, 5.94]
Δ preferred walking speed (km·h^− 1^)			0.881	[-1.62, 3.38]
Heart rate availability × Time point			-0.0198	[-0.0566, 0.0167]
Δ preferred walking speed × Time point			2.30	[-0.315, 4.92]

*LMM *linear mixed model, *PAEE *physical activity energy expenditure. *p ≤ 0.05, **p ≤ 0.01, ***p < 0.001

To support these longitudinal findings, Supplements 1 and 2 present LMM estimates of PAEE derived solely from ACC data, using the same model structure as in the primary analyses. In contrast to the primary model, where the interaction between change in walking speed and time point was not statistically significant, the ACC-only model showed a significant interaction (B = 2.14, *p* = 0.007; Supplement 2).

## Discussion

In this longitudinal study, we observed a decline in PAEE over the 4-year follow-up period in community-dwelling adults who were initially 75 to 85 years old. Older participants had slightly lower PAEE. However, after adjusting for preferred walking speed, baseline age was no longer associated with PAEE across timepoints during follow-up. The results suggest that walking speed may partly account for the association between age and PA, as preferred walking speed showed a strong cross-sectional association with PAEE. In the primary analyses, within-person change in walking speed was not associated with concurrent change in PAEE over time. The observed decline in PAEE over a 4-year period, equating to an average annual decrease of 4.3%, is consistent in direction but notably greater in magnitude than previous longitudinal findings based on accelerometry in community-dwelling older adults. In the Rush Memory and Aging Project (Rush MAP) a 2% annual decline among 519 participants was reported with a mean baseline age of ~ 82 years, over an average ~ 3.3 years of follow-up [3]. The Baltimore Longitudinal Study of Aging (BLSA) found an age-dependent decline in wrist-measured total activity counts, with an estimated 2% annual reduction at age 75 over a median 2.1-year follow-up among 499 older adults [30]. Both studies assessed PA as activity counts from wrist-worn accelerometers. The EPIC-Norfolk study, using hip-worn accelerometers found a larger, 3.2% annual decline in total activity counts over 6 years among participants with a mean baseline age of 67.8 years (*n* = 1259) [31]. In previous studies, participants at older ages experienced faster declines. For example, in Rush MAP, total PA of individuals aged 91 declined nearly twice as fast as PA of those aged 71, and in BLSA, the modeled annual rate of decline at age 75 (2%) was ten times greater than at age 65 (0.2%).

Across these cohorts, the present study showed the largest declines in total PA over time, followed by EPIC-Norfolk, while Rush MAP and BLSA reported smaller changes. These differences likely reflect how age was handled in statistical models. In the present study, Rush MAP and BLSA, baseline age was included as a fixed covariate, capturing between-person differences but not within-person aging effects. However, Rush MAP and BLSA also included an age × time interaction allowing older participants at baseline to decline faster which could produce smaller or more nuanced estimates of decline. These two cohorts also adjusted for multiple demographic and health covariates (e.g., sex, education, BMI, comorbidities), and notably, the BLSA models additionally controlled for preferred walking speed, which showed a significant cross-sectional association with PAEE in the present study. In the present study, annual decline in PAEE dropped to 3.9% after adjusting for preferred walking speed (Table 3, Model 3). The second-largest decline observed in EPIC-Norfolk was based on unadjusted estimates, unlike the multivariable-adjusted models used in the other cohorts. It is also noteworthy that the smallest declines in total PA were observed in cohorts using wrist-worn accelerometers, whereas EPIC-Norfolk used hip-worn devices and the present study combined thigh-worn ACC with HR monitoring. In a study by Skjødt et al. [32], thigh accelerometer placement provided the most accurate intensity classification in very old adults, suggesting that the present study’s thigh-based estimates may reveal a more pronounced true decline in PA than earlier wrist-based studies.

Even with these considerations, the present study demonstrates a steeper decline in PA than prior research, some of which may be attributable to the integration of HR data into the PAEE estimation. Including HR-based methods alongside ACC improves the precision of energy expenditure estimates, particularly in free-living conditions [17]. Moreover, applying individual calibration such as a walking test further improves accuracy by aligning the model with each person’s physiological response [17, 26]. Although HR availability was significantly associated with PAEE estimation (Table 3, Model 2), the decline in PAEE differed only by 1%-point from the unadjusted model. Furthermore, the non-significant interaction with time point suggests the influence of HR availability over PAEE remained stable across measurements. Thus, while HR might improve the accuracy of PAEE estimates, its availability does not appear to modify the observed rate of decline in PA over time. Importantly, when HR-based estimation was excluded and PAEE was assessed using ACC only, the estimated decline was lower, 2.9% per year (Supplement 1, Model 3), which is more consistent with prior research. Previous cross-sectional estimates of PA from our research group indicate that values based on HR monitoring tend to be higher than those derived from ACC data in older adults [33]. Together, these findings suggest that the integration of HR data may have uncovered a more pronounced decline in PAEE than would have been detected using ACC alone.

Preferred walking speed showed a strong association with PAEE in our cohort: each km·h^− 1^ faster walking speed corresponded to roughly 16% higher PAEE relative to the cohort’s mean. Both walking speed and PAEE declined over time; however, changes in walking speed were not clearly associated with changes in PAEE in the primary analyses. The non-significant interaction between walking speed and time point indicates that the strength of this association remained relatively consistent across measurement occasions. Similar cross-sectional findings have been reported where walking speed was robustly associated with total activity counts [30, 34].

Research on walking speed as a factor underlying change in PA during aging is scarce. To the best of our knowledge, only one study has comprehensively examined parallel changes over time of walking speed and monitored PA in older adults and assessed the associations between these measures over time. Yerrakalva et al. [13], using data from the EPIC-Norfolk cohort, reported that changes in preferred walking speed were associated with changes in total activity counts, with associations of a similar magnitude to those observed in the present study. However, direct comparisons are challenging, as participants in the EPIC-Norfolk cohort were on average about 10 years younger, the study used a shorter walking test protocol, and their models included a broader range of socioeconomic and health-related covariates. Analyses using accelerometer-derived PAEE alone were conducted as sensitivity analyses to facilitate comparison with prior studies relying on accelerometry-only estimates. These analyses indicated a statistically significant association, highlighting that observed change–change associations may depend on how PAEE is quantified rather than providing definitive evidence of a longitudinal relationship (Supplement 2). While preferred walking speed emerged as a meaningful correlate of PAEE, the analyses indicated a 3.9% annual decline in PAEE even after accounting for walking speed (Table 3, Model 3). This suggests that a substantial portion of the decline in activity may be driven by factors other than walking performance, such as behavioral adaptations [35] and environmental influences [36]. It is also possible that walking speed measured in the laboratory conditions did not fully capture changes in free-living walking performance. At follow-up, participants performed the walking test at a relatively higher intensity in relation to their declining physical capacity, as indicated by a small but significant increase in perceived exertion during the 6MWT. This is consistent with research showing that even usual-pace walking becomes subjectively more strenuous with aging and mobility decline [37], suggesting that the same laboratory task may represent a greater relative effort over time.

Consequently, laboratory walking tests may have overestimated free-living walking performance at follow-up, potentially underestimating the true decline in preferred walking speed and attenuating its longitudinal association with PA. These concerns highlight the importance of incorporating home or free-living walking assessments in future research, where pacing, environmental context, and motivational factors more closely mirror real-world mobility demands [38]. Nonetheless, preferred walking speed remains a powerful and conceptually important predictor when interpreting patterns of PA decline in older adults, particularly given that walking represents a major component of daily movement and is essential for maintaining functional independence.

Strengths of this study include the population-based sample and the longitudinal design. A notable strength is also the use of combined HR and ACC data to estimate PAEE. For most participants, PAEE estimation based on HR data were individually calibrated, and for the remaining participants, a group calibration equation based on all participants in the study with valid calibration was used. The combined HR and ACC method might provide a more physiologically grounded measure of PAEE compared to ACC alone. Additionally, the longitudinal design with repeated measures over a 4-year period enables the examination of within-person changes in PAEE and walking speed and thus provides valid information about changes occurring with increasing age.

This study has also limitations. First, the study population consisted of relatively healthy older adults, which truncates the distribution potentially leading to underestimation of associations. Although the sample was population-based, participants dropped out based on the availability of data, introducing selection bias. Moreover, individuals who agreed to participate in PA monitoring tend to be systemically healthier, have better functioning, and are more active than those who decline, as noted by Portegijs et al. [39] and this bias may be amplified in a longitudinal design. The study also relied on only two time points, which limits the ability to capture non-linear trajectories in the relationship between time, physical function, and PA. The assumption of linearity may therefore oversimplify the dynamics of change over time. In addition, the relatively small sample size and short follow-up period further constrain the ability to detect statistically significant associations between changes in PA and physical function.

## Conclusions

In conclusion, this study demonstrates that PAEE declines substantially with age among community-dwelling older adults. The observed decline was steeper than in previous reports, which may reflect the use of combined thigh-worn ACC and HR monitoring, potentially capturing intensity of activity more accurately than ACC alone. Preferred walking speed was strongly associated with PAEE at baseline; however, its change over time was not consistently associated with PAEE change in the primary analyses, suggesting that short-term declines in walking speed may not strongly translate into reductions in PAEE. These findings highlight the need for refined measurement approaches and for using walking speed to provide physiologically meaningful context when interpreting PA decline in older adults.

## Supplementary Information

Supplementary Material 1.

Supplementary Material 2.

## Data Availability

After completion of the study, data will be stored in the Finnish Social Science Data Archive without potential identifiers (open access). Until then, pseudonymized data is available to external collaborators upon reasonable request under the terms of data protection regulation. To request the data please contact the corresponding author.

## Abbreviations

1D CNN: One-dimensional convolutional neural network
6MWT: Modified six-minute walk test
ACC: Accelerometry
AGNES: Active Ageing – Resilience and External Support as Modifiers of the Disablement
B: Unstandardized regression coefficient
BLSA: Baltimore Longitudinal Study of Aging
BMI: Body mass index
ECG: Electrocardiography
EPIC-Norfolk: European Prospective Investigation into Cancer – Norfolk
HR: Heart rate
LMM: Linear mixed model
MAD: Mean amplitude deviation
MMSE: Mini-Mental State Examination
PA: Physical activity
PAEE: Physical activity energy expenditure
RPE: Rate of perceived exertion
Rush MAP: Rush Memory and Aging Project
SPPB: Short Physical Performance Battery
SD: Standard deviation
