# Supplementary material for: Longitudinal associations between physical activity and preferred walking speed in community-dwelling older adults: a 4-year follow-up study
Source: Eur Rev Aging Phys Act. 2026 Jun 4;23:30. doi: 10.1186/s11556-026-00419-9 (PMC13464384; doi:10.1186/s11556-026-00419-9)
Supplement: Supplementary file 1 [file 11556_2026_419_MOESM1_ESM.docx]

**Supplement 1.** LMM estimates of age, time, sex, and preferred walking speed on ACC-based PAEE (n = 282-299)

| Independent variables | | Estimate | 95% confidence interval |
| --- | --- | --- | --- |
| Model 1 (Number of observations = 598, *n* = 299) | | | |
| (Intercept) | | 29.0*** | [28.1, 29.8] |
| Baseline age (year) | | -0.319* | [-0.568, -0.0705] |
| Time point (reference = baseline) | |  |  |
|  | Follow-up | -3.36*** | [-4.02, -2.71] |
| Model 2 (Number of observations = 556, *n* = 287) | | | |
| (Intercept) | | 27.5*** | [26.5, 28.6] |
| Baseline age (year) | | -0.343** | [-0.585, -0.101] |
| Time point (reference = baseline) | |  |  |
|  | Follow-up | -3.36*** | [-4.02, -2.71] |
| Sex (reference = women) | |  |  |
|  | Men | 3.32*** | [1.76, 4.88] |
| Wear time (day) | | -0.237 | [-0.778, 0.304] |
| Model 3 (Number of observations = 516, *n* = 282) | | | |
| (Intercept) | | 28.3*** | [27.4, 29.3] |
| Baseline age (year) | | -0.0194 | [-0.246, 0.206] |
| Time point (reference = baseline) | |  |  |
|  | Follow-up | -2.87*** | [-3.56, -2.17] |
| Sex (reference = women) | |  |  |
|  | Men | 1.92** | [0.499, 3.34] |
| Wear time (day) | | -0.231 | [-0.773, 0.311] |
| Preferred walking speed (km/h) | | 3.94*** | [2.99, 4.88] |
| Preferred walking speed × Time point | | -0.215 | [-1.16, 0.726] |

LMM = linear mixed model, ACC = accelerometry, PAEE = physical activity energy expenditure. *p ≤ 0.05, **p ≤ 0.01, ***p < 0.001.
