# Supplementary material for: Longitudinal associations between physical activity and preferred walking speed in community-dwelling older adults: a 4-year follow-up study
Source: Eur Rev Aging Phys Act. 2026 Jun 4;23:30. doi: 10.1186/s11556-026-00419-9 (PMC13464384; doi:10.1186/s11556-026-00419-9)
Supplement: Supplementary file 2 [file 11556_2026_419_MOESM2_ESM.docx]

**Supplement 2.** LMM estimates of change in preferred walking speed ACC-based PAEE (n = 252)

| Independent variables | | Estimate | 95% confidence interval |
| --- | --- | --- | --- |
| Model 4 (Number of observations = 486, *n* = 252) | | | |
| (Intercept) | | 28.9*** | [27.8, 29.9] |
| Baseline age (year) | | 0.0474 | [-0.201, 0.296] |
| Time point (reference = baseline) | |  |  |
|  | Follow-up | -3.32*** | [-4.01, -2.63] |
| Sex (reference = women) | |  |  |
|  | Men | 1.25 | [-0.251, 2.75] |
| Wear time (day) | | -0.219 | [-0.764, 0.327] |
| Baseline preferred walking speed (km·h^-1^) | | 4.20*** | [3.09, 5.30] |
| Δ preferred walking speed (km·h^-1^) | | 1.63 | [-0.216, 3.48] |
| Δ preferred walking speed × Time point | | 2.14** | [0.608, 3.68] |

LMM = linear mixed model, ACC = accelerometry, PAEE = physical activity energy expenditure. *p ≤ 0.05, **p ≤ 0.01, ***p < 0.001.
